# Effects of Periodization Core Training on Physical Fitness in College Table Tennis Players

**DOI:** 10.1371/journal.pone.0323430

**Published:** 2025-05-15

**Authors:** Kuan Dong, Guyeol Jeong, Jing Tian, Buongo Chun

**Affiliations:** 1 College of Physical Education and Sports, Central China Normal University, Wuhan, China; 2 Department of Physical Education, Chosun University, Gwangju, Republic of Korea,; 3 Graduate School of Physical Education, Myongji University, Yongin, Republic of Korea; University of Tehran, IRAN, ISLAMIC REPUBLIC OF

## Abstract

**Objective:**

This study aimed to analyze the effects of 12-week periodized core training on the physical fitness of college table tennis players.

**Methods:**

A randomized controlled experimental design was employed, and 18 college table tennis players (male = 11, female = 7) were randomly assigned to the core training group (CT, n = 9) and the control group (CON, n = 9). The core training group performed periodized core training for 12 weeks. All variables were assessed at three time points: pre-test, mid-test (after 9 weeks), and post-test.

**Results:**

Significant interactions were found between time and group for muscle endurance, balance, and agility, as demonstrated in Left Side Bridge (p < 0.05), Right Side Bridge (p < 0.001), Plank (p < 0.01), and Edgren Side Step (p < 0.001). However, no significant interactions were observed for speed, muscle strength, or power.

**Conclusion:**

Periodized core training has a positive effect on the muscle endurance, agility, and balance of college table tennis players. The improvement in agility may be attributed to the integration of sport-specific periodized training. Further research is required to examine its effects on speed and anaerobic capacity. Periodized core training appears to have limited effects on strength and power, suggesting it can be used as a supplementary element within a comprehensive training program to enhance physical fitness and performance among college table tennis players.

## Introduction

Table tennis, an Olympic sport, currently engages approximately 300 million participants worldwide, with around 40 million competitive players [1,2]. Changes to the regulations and equipment, including the introduction of the 11-point scoring system, the adoption of 40 + seamless balls, and the prohibition of celluloid-based adhesives, have transformed the sport into a more dynamic and spectator-friendly activity [3]. These changes have subsequently increased the demands for rapid response time and explosive power from players [3].

As a high-intensity intermittent sport [4], table tennis exhibits a unique energy system distribution: 96.5% oxidative system, 2.5% phosphagen system, and 1% glycolytic system. The phosphagen system plays a crucial role due to the repetitive nature of brief actions lasting less than 4 seconds [5]. As competition levels increase, rallies become more prolonged, shifting the emphasis toward the glycolytic system and enhancing the importance of anaerobic endurance [2,6]. These physiological characteristics underscore the essential requirement for robust core strength and stability among table tennis players.

Contemporary table tennis ranks among the fastest ball sports, with ball velocities reaching 120 km/h in high-level matches and occasionally peaking at 160 km/h [7]. Players execute more than 30 strokes per minute, with each stroke lasting less than 4 seconds and rest intervals within 15 seconds [1]. During continuous rallies, players must constantly accelerate, decelerate, and change direction while seeking optimal striking positions. In this context, core strength plays a fundamental role in maintaining postural stability and enabling efficient movement execution [8–10]. The performance of table tennis players is significantly influenced by both technical and physical fitness components. Research has demonstrated that physical attributes such as grip strength, agility, leg strength, explosive power, directional change capability, and speed directly impact technical execution in table tennis [11,12]. Notably, core strength and stability serve as the foundation for these physical attributes, facilitating power generation, balance maintenance, and precise movement execution. With the continuous evolution and development of table tennis, the increasing intensity and pace of matches have led coaches to recognize the importance of physical training [13].

Previous studies have demonstrated the effectiveness of core training in enhancing muscular endurance and balance; however, findings regarding its impact on agility and speed have been inconsistent [14–17]. Prieske (2016) reported that core training showed a moderate effect size (ES = 0.71) in improving athletic performance. Nevertheless, most existing research has been limited to either stabilization phases [18–20] or strength development phases [21,22], lacking the integration of sport-specific movements and power development in the transformation phase.

Optimal physiological adaptation requires a periodized training program lasting approximately 12 weeks, with each goal-oriented phase maintaining consistency for 3–4 weeks. Additionally, a training frequency of 2–4 sessions per week with 24–48 hours of recovery time is recommended [23,24]. Considering these principles and the limitations of existing research, it is anticipated that a periodized core training program incorporating sport-specific elements would be more effective in enhancing the physical fitness of table tennis players.

Therefore, this study aims to analyze the effects of a 12-week periodized core training program on the physical fitness of collegiate table tennis players. Unlike previous research, this study systematically incorporates stabilization, strength, and power development phases while integrating table tennis-specific movements, potentially offering a more effective training methodology. The findings of this study may provide valuable insights for developing more comprehensive and sport-specific training programs for table tennis players across various competitive levels.

## Methods

### Participants

The required sample size for this study was pre-estimated using G*Power 3.1 software (Dusseldorf, Germany). The “ANOVA: Repeated measures, within-between interaction” option was selected, with power, α, and effect size set at 0.95, 0.05, and 0.6, respectively. The effect size was referenced from Dong (2023) [25], indicating that the comprehensive effect size of core training on athletic performance ranges from 0.32 to 0.90, with an average effect size of 0.59. The estimation results showed that the minimum sample size required for this study was 10 participants. Considering a potential dropout rate of 20% [26,27], at least 12 participants were needed. Based on this, the study recruited 18 athletes to complete all tests in the experiment.

The subjects were randomly assigned into a core training group (CT, n = 9) and a control group (CON, n = 9) using a computer-generated random number table (Table 1). All table tennis athletes had participated in national competitions and were familiar with the CT intervention. The eligibility criteria for this study were: (1) at least 10 years of table tennis training and competition experience; (2) continuous table tennis training for at least six months prior to inclusion in the study; (3) no core training experience in the past three months; (4) no participation in any other competitive sports activities; (5) no injuries in the past month. The study adhered to ethical standards outlined in the Declaration of Helsinki and was approved by the Institutional Ethical Review Board at the Myongji University, with reference code 2023-03-001-001. Written informed consent was obtained from all participants involved in the study. April 30, 2023 pre-study measurements were completed. CT program was implemented between the dates May 1, 2023- July 28, 2023. July 1, 2023 mid-study measurements were repeated. July 31, 2023 post-study measurements were completed.

**Table 1:** Descriptive statistics of the participants.

Group	Age (years)	Body mass (kg)	Height (cm)	Experience (years)
CT (*n* = 9)	20.22 ± 1.09	64.56 ± 9.91	172.67 ± 8.25	13.33 ± 2.06
CON (*n* = 9)	20.11 ± 1.05	68.67 ± 11.09	174.78 ± 10.32	11.67 ± 2.60
*P*	.829	.638	.419	.151

CT: core training group; CON: control group.

### Study design and experimental approach

This study employed a randomized clinical trial (RCT) design with evaluators blinded to the group assignments. Before the first test, all subjects underwent a familiarization session with the testing tasks, followed by a pre-test assessment of physical fitness indicators (Pre-test). A mid-test was conducted before the start of the sport-specific transformation phase, and a post-test was conducted after the transformation phase. The control group did not receive any intervention. The tests were completed over two days, both in the afternoon to control for the influence of diurnal performance variations. The testing followed a specific order: warm-up (4 minutes of slow jogging and 3 minutes of dynamic stretching), Grip, Y-Balance, Edgren Side Step, Plank and Side Bridge test on the first day. On the second day, the tests included the Standing Long Jump (SLJ), Countermovement Jump (CMJ), 30m sprint, and 400m test (Table 2). Subjects were instructed to avoid strenuous physical activities, maintain their usual diet for 24 hours before the tests, and abstain from caffeine and alcohol consumption [28,29]. The training began 72 hours after the completion of the Pre-test measurements, and subsequent Mid-test and Post-test assessments were conducted 72 hours after the end of the training phases, under the same conditions and in the same testing order. All participants performed the tests in a fixed order over two days. To mitigate fatigue effects on subsequent tests, sufficient rest periods were incorporated between assessments, and tests likely to induce fatigue were scheduled last.

**Table 2:** Study procedure.

Study duration (weeks)		
Week 1	Pre-test	
	Monday	Tuesday
	Grip, Balance, Edgren Side Step, Endurance	SLJ, CMJ, 30m, 400m
Week 2–4	Core stability training	
	Elbow plate, Side bridge, YTA-shaped, Single side bridge	
Week 5–7	Core strength training	
	Push up, Wipers, Pull out band, Side step slide	
Week 8–10	Core power training	
	Medicine Ball Slam, Medicine Ball, Surrounding, Change-leg jump	
Week 11	Mid-test	
Week 12–14	Core sport-specific training	
	Multi-directional slide, Rope Pull, Medicine Ball Throwing	
Week 15	Post-test	

### Training protocols

**Fig 1:**
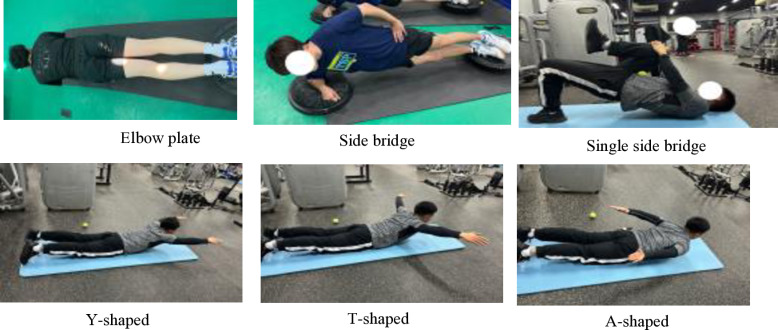
Core stability training.

**Fig 2:**
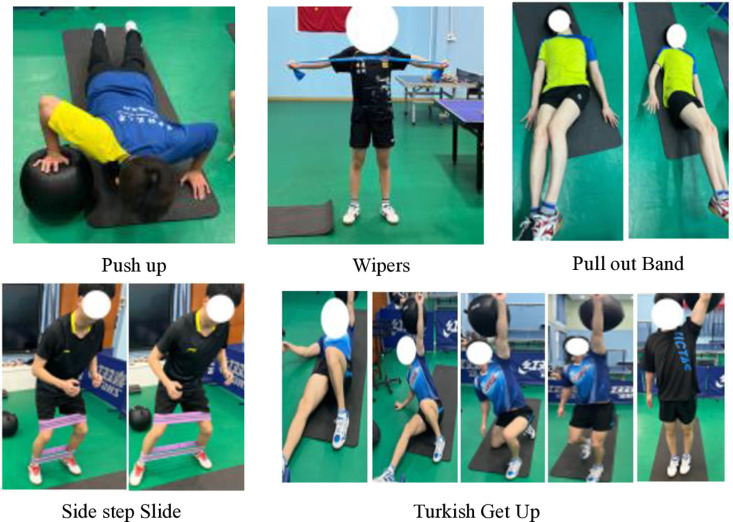
Core strength training.

**Fig 3:**
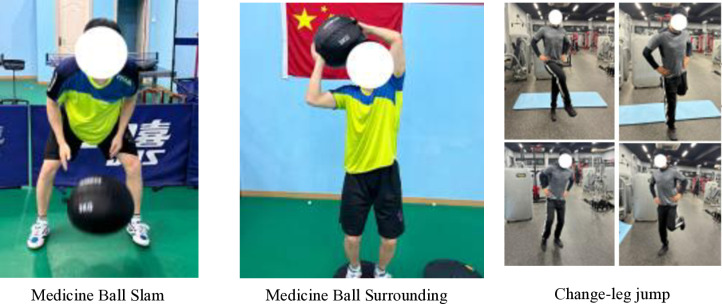
Core power training.

**Fig 4:**
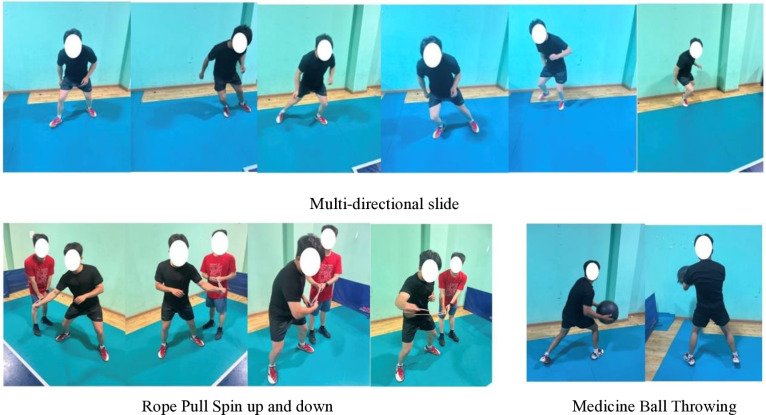
Core sport-specific training.

Periodization can be defined as a logical sequential, phasic method of manipulating fitness and recovery phases to increase the potential for achieving specific performance goals while minimizing the potential for nonfunctional over-reaching, overtraining, and injury [30]. On the other hand, programming deals with the micromanagement of the training process and deals with exercise selection, volume, intensity, etc [30]. From a practical application perspective, the effectiveness of core training should ultimately manifest in the improvement of sport-specific technical skills. To achieve this, it is essential to identify the primary core muscle groups based on the characteristics of the sport and to enhance muscle functions that align with sport-specific technical movements [25]. The periodized core training program was designed based on previous studies [18,20–22,25,31–33]. And the core training program in this study was divided into stability, strength, power, and sport-specific transformation phases (Table 3, Figs 1–4). The core training group participated in three training sessions per week for 12 weeks (Monday, Wednesday, and Friday). In 12-week periodized core training program, we strategically selected exercises to enhance various aspects of motor performance, including balance and stability. For example, during the core stability phase (Weeks 1–3), exercises such as the plank (elbow plate hold) and side bridges were incorporated specifically to develop athletes’ balance and postural control. These foundational exercises, performed for 40 seconds and 30 seconds per side respectively (with 12 repetitions for single side bridges and YTA-shaped movements), served to enhance muscular endurance and stability. As the program progressed to the core strength phase (Weeks 4–6), dynamic movements like push-ups and Turkish Get-Ups were introduced not only to build maximum muscular strength but also to challenge and further improve balance through controlled movement patterns. In subsequent phases, the emphasis shifted towards explosive power and sport-specific skills; however, each phase continued to incorporate elements that promote balance and coordination, ensuring that the training adaptations are effectively transferred to performance on the playing field (Table 3). The control group did not receive intervention. The training sessions followed a circuit training methodology.

**Table 3:** Core training programs.

Week	Phase	Objective	Exercise Interventions	Frequency, Sets, Rest
Week 1-3	Core stability training	Muscular endurance (Tissue Adaptation Phase)	Elbow plate 40s, Side bridge 30s/side, Single side bridge 12 reps, YTA-shaped 12 reps.	3 sessions/week, 3 sets, 30s.
Week 4-6	Core strength training	Muscular Strength (Maximum Muscular Strength Phase)	Push up 15 reps/side, Wipers 5reps/side, Pull out Band 10 reps, Side step Slide 5 steps/side, Turkish Get Up 10 reps.	3 sessions/week, 3 sets, 30s.
Week 7-9	Core power training	Power, agility (explosive power conversion phase	Medicine Ball Slam 8 reps, Medicine Ball Surrounding 8 reps, Change-leg jump 8 reps/side.	3 sessions/week, 3 sets, 90s.
Week 10-12	Core sport-specific training	Sport-specific performance (maintenance phase)	Multi-directional slide 10 reps/side, Rope Pull (Spin up and down) 10 reps/side, Medicine Ball Throwing 10eps.	3 sessions/week, 3 sets, 90s.

### Physical fitness assessments

#### Anthropometric measurements.

The measurement of height was completed using a stadiometer (Bodymeter, SECA, Germany, with the nearest 0.1 cm) and body weight using an electronic scale (Body Complete, Beurer, Germany, with the nearest 0.1 kg).

#### Muscle strength.

Muscle strength was assessed using the Hand Grip Test, which has been validated as a reliable indicator of muscle strength [34,35]. The testing protocol followed the methods [36,37], utilizing a handheld dynamometer (Takei 5101, Takei Instruments Ltd., Tokyo, Japan) to measure the maximum grip strength of the dominant hand. The dynamometer was adjusted to fit the size of each participant’s hand before testing. Participants were instructed to hold the dynamometer with their arm parallel to their body, maintaining a downward position without wrist flexion. Each participant performed three maximum voluntary contractions of 3–5 seconds each, with a 30-second rest interval between attempts. The best result (measured in kg) was used for the data analysis.

#### Power.

Power was measured using the Standing Long Jump (SLJ) and Countermovement Jump (CMJ) tests, both of which are widely recognized for their reliability in assessing explosive power [38]. The CMJ reflects vertical displacement capability, while the SLJ measures horizontal displacement capability. 1) SLJ: The testing protocol followed the methods [39,40]. Participants started from a standing position, swinging their arms and bending their knees to jump forward as far as possible. They were required to land on both feet simultaneously without falling forward or backward. The horizontal distance from the take-off line to the nearest heel was measured using a metric tape measure (Model SQ8188, Chaoqun, China). Each participant performed three trials with a 15-second rest interval between attempts, and the longest distance (measured in cm) was used for the data analysis. 2) CMJ: The testing protocol followed the methods [41,42]. Participants performed a rapid squat to a 90° knee bend from a standing position and then jumped as high as possible, attempting to reach the highest vane on a jump mat (CSSIT, China). Each participant performed three trials with a 10-second rest interval between attempts, and the highest jump (measured in cm) was used for the data analysis.

#### Muscular endurance.

Muscular endurance was measured using the Plank and Side Bridge tests, both of which are recognized for their high reliability in assessing muscular endurance [43]. The testing protocol followed the methods [43]. 1) Side Bridge Test: Participants lay on their side with legs straight and elbow bent at 90°. They lifted their pelvis off the ground, forming a straight line with their body, while placing the opposite hand on their hip. The test ended when they could no longer maintain the position. 2) Plank Test: Participants maintained the plank position for as long as possible, with the test ending when they could no longer hold the position. Each participant performed one trial, and the time (measured to 0.01 seconds) was used for the data analysis.

#### Speed.

Speed is a critical performance factor in modern table tennis [44]. It was measured using the 30m Sprint Test. A straight track marked with a start and finish line was used. The testing protocol followed the methods [45]. Participants prepared at the start line and sprinted to the finish line at the signal of a professional athletics coach. The time was recorded using a stopwatch, accurate to 0.01 seconds. Each participant performed two trials with a 3-minute rest interval between attempts, and the best time (measured in seconds) was used for analysis.

#### Agility test.

Agility was measured using the Edgren Side Step Test, known for its high reliability in assessing agility [46]. The testing protocol followed the methods [46,47]. Five cones were placed in a straight line 1 meter (3.28 feet) apart. Participants stood straddling the center cone, facing forward. They moved laterally to the right and left, crossing the outermost cone with the corresponding foot. The number of cones crossed in 10 seconds was recorded. Each participant performed two trials with a minimum of 3 minutes rest between attempts to ensure full recovery. The highest number of crosses was used for data analysis.

#### Balance test.

Balance was measured using the Y-Balance Test (YBT), which has high reliability [48]. The testing protocol followed the methods [48]. Participants stood barefoot on one leg on the YBT device, pushing the test plate as far as possible in the anterior (A), posteromedial (PM), and posterolateral (PL) directions with the toes of the non-support leg. Each direction was measured three times, and the maximum distance (measured in cm) was recorded for analysis.

#### Anaerobic capacity.

Anaerobic capacity was measured using the 400m Test, following the protocol [49]. After a thorough warm-up, participants prepared at the start line and ran 400m as fast as possible at the signal of a professional athletics coach. The time was recorded using a stopwatch, accurate to 0.01 seconds. Each participant performed two trials with a minimum of 5 minutes rest between attempts to ensure full recovery. The best time (measured in seconds) was used for analysis.

### Statistical analysis

The results are presented as means ± standard deviation (SD). All the variables presented a normal distribution according to the Shapiro-Wilk test. Two way ANOVA with the Bonferroni post hoc test was used to test for differences between time points and between groups. If the data violated Mauchly’s sphericity assumption, the Greenhouse-Geisser correction was applied. The effect size was measured using partial eta squared (η_p_^2^). The effect sizes were evaluated against the following thresholds: 0.04 for minimum effect size, 0.25 for moderate effect size, and 0.64 for strong effect size [50]. The statistical analysis was carried out using SPSS (version 28.0.0.0, IBM, USA). The upper limit for statistical significance was set at p < 0.05.

## Results

Descriptive statistics of physical fitness outcomes pre, mid and post-intervention can be observed in Table 4.

**Table 4:** Changes in physical fitness outcomes (Mean ± SD).

Physical fitness	Variable	Group	Pre-test	Mid-test	Post-test	Between-groups analysis
Muscle Strength	Grip (kg)	CT	36.44 ± 9.90	36.44 ± 9.09	36.01 ± 9.14	F = 0.314; p = 0.733; η_p_^2 ^= 0.019
		CON	36.73 ± 10.66	35.97 ± 11.05	35.64 ± 11.15	
Power	CMJ (cm)	CT	39.21 ± 8.21	39.66 ± 7.46	39.84 ± 7.69	F = 0.899; p = 0.417; η_p_^2 ^= 0.053
		CON	38.84 ± 8.70	38.80 ± 8.13	38.72 ± 8.42	
	SLJ (cm)	CT	216.56 ± 38.68	220.22 ± 41.14	220.67 ± 39.85	F = 0.873; p = 0.427; η_p_^2 ^= 0.052
			CON	216.11 ± 38.33	215.22 ± 37.75	214.33 ± 37.73
Muscular endurance	Bridge L (s)	CT	43.72 ± 14.76	63.53 ± 14.89 ^***^^,^^‡^	70.46 ± 22.64 ^***^^,^^‡‡^	F = 5.329; p < 0.05; η_p_^2 ^= 0.25
		CON	38.11 ± 9.33	44.15 ± 10.93	47.58 ± 12.30	
	Bridge R (s)	CT	53.05 ± 16.45	76.74 ± 26.11 ^***^^,^^‡^	82.47 ± 21.92 ^***^^,^^‡‡^^,^^†^	F = 13.351; p < 0.001; η_p_^2 ^= 0.455
			CON	46.18 ± 10.04	50.61 ± 9.92	52.96 ± 9.58
	Plank (s)	CT	87.78 ± 24.26	121.19 ± 40.94 ^***^^,^^‡^	132.62 ± 36.90 ^***^^,^ ^‡‡^^,^ ^†††^	F = 9.052; p < 0.01; η_p_^2 ^= 0.361
			CON	64.86 ± 23.03	78.02 ± 23.11	80.47 ± 25.74
Speed	30m (s)	CT	5.63 ± 0.97	5.22 ± 0.68^**^	5.09 ± 0.69^**^	F = 1.995; p = 0.153; η_p_^2 ^= 0.111
		CON	5.94 ± 0.97	5.75 ± 0.90	5.72 ± 0.84	
Agility	Edgren sidestep (number)	CT	29.78 ± 3.56	30.33 ± 3.24	33.22 ± 3.11 ^***^^,^ ^‡^^,^ ^†††^	F = 12.487; p < 0.001; η_p_^2 ^= 0.438
		CON	29.89 ± 2.93	29.11 ± 2.76	29.44 ± 3.32	
Anaerobicapacity	400m (s)	CT	84.30 ± 19.23	79.13 ± 15.08^*^	79.11 ± 15.61^*^	F = 1.758; p = 0.203; η_p_^2 ^= 0.099
		CON	93.76 ± 17.22	92.83 ± 14.04	92.35 ± 13.75	
Balance	YBT Left-A (cm)	CT	61.33 ± 12.72	73.11 ± 12.00^***^	74.78 ± 12.54 ^***^^,^ ^†^	F = 21.683; p < 0.001; η_p_^2 ^= 0.575
		CON	63.33 ± 12.36	65.33 ± 14.82	65.78 ± 13.71	
	YBT Left-PM (cm)	CT	90.78 ± 6.40	93.11 ± 5.33^*^	100.67 ± 7.84 ^***^^,^ ^‡^^,^ ^†††^	F = 6.226; p < 0.05; η_p_^2 ^= 0.280
			CON	83.78 ± 14.54	84.33 ± 15.44	87.78 ± 7.77
	YBT Left-PL (cm)	CT	90.00 ± 12.77	93.78 ± 9.88	104.33 ± 9.12 ^***^^,^ ^‡‡‡^^,^ ^†††^	F = 8.134; p < 0.01; η_p_^2 ^= 0.337
			CON	86.22 ± 12.31	86.67 ± 10.99	87.78 ± 7.77
	YBT Right-A (cm)	CT	58.22 ± 9.92	70.78 ± 8.04^***^	71.11 ± 7.69^***^	F = 20.547; p < 0.001; η_p_^2 ^= 0.562
			CON	62.89 ± 13.93	62.44 ± 13.19	61.56 ± 11.83
	YBT Right-PM (cm)	CT	82.33 ± 10.51	94.78 ± 7.68 ^***^^,^ ^‡‡‡^	99.22 ± 7.17 ^***^^,^ ^‡‡^^,^ ^††^	F = 31.677; p < 0.001; η_p_^2 ^= 0.664
		CON	83.78 ± 11.51	81.33 ± 11.00	80.78 ± 10.94	
	YBT Right-PL (cm)	CT	93.44 ± 9.89	101.89 ± 7.49^***^	104.44 ± 6.60 ^***^^,^ ^‡^^,^ ^†^	F = 27.714; p < 0.001; η_p_^2 ^= 0.634
		CON	94.44 ± 12.03	92.78 ± 12.12	92.22 ± 14.30	

YBT: Y-Balance Test; A: Anterior; PM: Posteromedial; PL: Posterolateral;

*Significant difference compared with pre-test, p < 0.05,

**p < 0.01;

***p < 0.001;

†Significant difference compared with mid-test, p < 0.05,

††p < 0.01,

†††p < 0.001;

‡Significant difference between groups, p < 0.05,

‡‡p < 0.01,

‡‡‡p < 0.001.

### Muscle Strength and Power

There was not a significant main effect of time on grip, CMJ and SLJ. And no significant interactions between time and groups were found in grip, CMJ and SLJ.

### Muscle Endurance

For the muscle endurance, significant interactions between time and groups were found in Side Bridge L (F = 5.329; p < 0.05; η_p_^2^ = 0.25), Side Bridge R (F = 13.351; p < 0.001; η_p_^2^ = 0.455), Plank (F = 9.052; p < 0.01; η_p_^2^ = 0.361). Post-hoc test showed that the core training group improved significantly from pre-test to mid-test and from mid-test to post-test (p < 0.05). Furthermore, significant differences between the core training and control groups were found at mid-test (p < 0.05) and post-test (p < 0.01).

### Speed

For the 30m sprints, no significant interactions between time and groups were found. Post-hoc test showed that the core training group improved significantly from pre-test to mid-test and from mid-test to post-test (p < 0.01). However, no significant differences between the core training and control groups were found at mid-test and post-test.

### Agility

For the agility, significant interactions between time and groups were found in Edgren Side Step (F = 12.487; p < 0.001; η_p_^2^ = 0.438). Post-hoc test showed that the core training group improved significantly from mid-test to post-test (p < 0.05). However, significant differences between the core training and control groups were found at post-test (p < 0.01).

### Balance

For the balance, significant interactions between time and groups were found in Left-A (F = 21.683; p < 0.001; η_p_^2^ = 0.575), Left-PM (F = 6.226, p < 0.05; η_p_^2^ = 0.280), Left-PL (F = 8.134, p < 0.01; η_p_^2^ = 0.337). And significant interactions between time and groups were found in Right-A (F = 20.547, p < 0.001; η_p_^2^ = 0.562), Right-PM (F = 31.677; p < 0.001; η_p_^2^ = 0.664), Right -PL (F = 27.714; p < 0.01; η_p_^2^ = 0.634). Post-hoc tests showed that the core training group improved significantly from pre-test to mid-test and from mid-test to post-test (p < 0.05). However, significant differences between the core training and control groups were found at post-test (p < 0.01).

### Anaerobic Capacity

For the anaerobic capacity, no significant interactions between time and groups were found in 400m. Post-hoc test showed that the core training group improved significantly from pre-test to mid-test and from mid-test to post-test (p < 0.05). However, no significant differences between the core training and control groups were found at mid-test and post-test.

## Discussion

The present study investigated the effects of a 12-week periodized core training program on physical fitness components in table tennis athletes. The findings demonstrated significant improvements in core muscular endurance, balance, and agility, while the effects on speed and anaerobic capacity showed mixed results. Notably, no significant improvements were observed in strength and explosive power measures.

The significant enhancement in muscle endurance observed in this study aligns with previous research findings in various athletic populations. Dehnou (2020) reported substantial improvements in core muscle endurance among elite Greco-Roman wrestlers following a 4-week core strength training program, while Sever and Zorba (2018) documented similar improvements in soccer players after an 8-week intervention. At the 9-week mid-test, muscle endurance improved significantly relative to baseline levels. The improvements in core endurance can be attributed to the progressive nature of our periodized program, which likely facilitated enhanced neuromuscular adaptation, as evidenced by improved performance in both the Side Bridge and Plank tests. This adaptation is particularly relevant for table tennis players, who must maintain stable postures during rapid rotational movements [32].

Our findings regarding balance ability correspond with previous studies that demonstrated improved postural control following core training interventions. Granacher (2014) [51] reported enhanced balance in adolescents after a 6-week program, while Park (2016) observed similar improvements in archery athletes following a 12-week intervention. At the 9-week mid-test, balance ability improved significantly relative to baseline levels. The enhancement in balance performance can be explained through optimized neuromuscular control mechanisms, particularly the reduction in spinal reflex excitability during postural tasks [52]. The sport-specific nature of our training program’s transformation phase may have enhanced the transfer of these improvements to table tennis-specific movements, which is crucial given the sport’s demand for precise balance control during both defensive and offensive maneuvers.

The improvement in agility observed in our study presents an interesting contrast to previous research. While several studies reported no significant changes in agility following core training [15,53], our results showed meaningful improvements. This discrepancy may be attributed to three key factors in our study design: the periodized nature of the program, the incorporation of sport-specific movements in the transformation phase, and the longer intervention duration. The enhanced agility performance is particularly relevant for table tennis players, as it directly impacts their ability to execute rapid, multi-directional movements essential for effective court coverage and stroke execution.

Regarding speed development, our results partially align with previous research findings. Prieske (2016) and Hoshikawa (2013) [54] reported improvements in sprint performance following core training interventions in youth soccer players. However, contrasting results were reported by Parkhouse and Ball (2011) [55], who found no significant improvements in sprint performance following a 6-week program. These divergent findings might be explained by differences in training duration, population characteristics, and training specificity. Our 12-week intervention period may have provided sufficient time for adaptation, while the integration of sport-specific movements likely enhanced the transfer of training effects to performance outcomes.

The improvements in anaerobic capacity observed in our study support previous research findings. Soslu (2019) [56] documented significant improvements in peak power and mean power following an 8-week core training program in basketball players, while Koçak (2022) [57] reported similar enhancements in soccer players. These improvements can be attributed to enhanced force production and transmission through a more stable core, coupled with improved neuromuscular efficiency leading to reduced energy expenditure during high-intensity movements. This adaptation is particularly beneficial for table tennis players, who rely heavily on anaerobic energy systems during intense rallies and critical match points.

The absence of significant improvements in strength and power measures aligns with previous research findings in trained athletes. Stanton (2004) [58] reported improvements in trunk stability but no changes in athletic performance following a 6-week core training program in adolescent athletes. Similarly, Prieske (2015) [59] found no significant increase in maximal isometric force of trunk flexors in elite soccer players after a 9-week progressive core strength training program. This lack of improvement can be explained by several factors. First, the bodyweight-based core exercises may not have provided sufficient stimulus to induce significant strength gains in trained athletes, as traditional core training typically fails to exceed 10% of maximum spinal flexion intensity [60]. Second, the concept of adaptive reserve suggests that trained athletes have less potential for strength improvements compared to novices [61]. Additionally, Prieske (2015) found only moderate correlations between trunk and lower limb muscle activation during jumping tasks, suggesting limited transfer between core stability and lower limb power production.

The findings of this study have important implications for training program design and implementation in table tennis. The significant improvements in core endurance, balance, and agility suggest that periodized core training can effectively enhance these specific aspects of physical fitness. The integration of sport-specific movements in the latter phases of training appears to be crucial for optimizing training outcomes, particularly for sport-specific adaptations. However, the limited effects on strength and power suggest that additional training methods may be necessary to develop these qualities effectively in table tennis players.

### Limitations

First, the use of a mixed-gender sample, while representative of the sport, may mask potential gender-specific responses to core training. Second, the study primarily focused on physical fitness outcomes without directly measuring table tennis-specific performance parameters, such as stroke accuracy, movement efficiency, and match statistics. Third, the lack of biomechanical data—such as kinematic analyses of table tennis-specific movements, electromyographic assessments of muscle activation patterns, and force platform measurements during power-based activities—restricts our understanding of the mechanisms underlying the observed performance improvements.

## Conclusion

In conclusion, this study provides evidence that a 12-week periodized core training program can effectively enhance core muscular endurance, balance, and agility in table tennis players. While the program showed promising effects on speed and anaerobic capacity, its limited impact on strength and power suggests the need for additional training methods to develop these qualities. The successful integration of sport-specific movements and proper periodization appears crucial for optimizing training outcomes. These findings provide valuable guidance for coaches and practitioners in developing comprehensive training programs for table tennis players.

## Supporting information

S1 Table Physical fitness data. (PDF)
