# Supplementary material for: Effects of Periodization Core Training on Physical Fitness in College Table Tennis Players
Source: PLoS One. 2025 May 15;20(5):e0323430. doi: 10.1371/journal.pone.0323430 (PMC12080838; doi:10.1371/journal.pone.0323430)
Supplement: S1 Table — (PDF) [file pone.0323430.s001.pdf]

Group	Grip1	Grip2	Grip3	CMJ1	CMJ2	CMJ3	SLJ1	SLJ2
1.00	41.50	43.10	40.40	36.60	36.97	34.50	225.00	236.00
1.00	41.60	40.10	40.60	38.40	39.00	39.20	210.00	204.00
1.00	48.70	47.80	47.50	47.60	47.70	47.80	265.00	278.00
1.00	40.40	37.70	37.50	41.00	41.20	42.00	235.00	240.00
1.00	42.90	44.60	43.50	55.00	54.00	55.20	275.00	280.00
1.00	41.50	39.30	40.70	40.00	40.50	40.20	205.00	205.00
1.00	27.20	27.40	27.30	35.30	35.00	35.50	203.00	199.00
1.00	21.30	22.10	20.40	31.00	30.60	31.20	176.00	170.00
1.00	22.90	25.90	26.20	28.00	32.00	33.00	155.00	170.00
2.00	45.50	45.10	45.00	48.80	49.20	49.50	250.00	250.00
2.00	48.50	48.00	47.80	48.90	47.10	47.60	253.00	251.00
2.00	45.70	45.30	44.70	42.90	41.00	41.70	240.00	241.00
2.00	48.00	47.90	47.70	43.20	44.00	43.20	245.00	244.00
2.00	31.10	30.90	30.80	44.50	44.00	43.90	250.00	245.00
2.00	22.70	21.60	20.60	27.50	27.90	27.50	162.00	160.00
2.00	20.60	20.20	20.20	28.30	28.80	28.00	170.00	172.00
2.00	30.00	29.80	29.10	36.20	36.80	36.90	189.00	190.00
2.00	35.50	34.90	34.90	29.30	30.40	30.20	186.00	184.00

SLJ3	Plank1	Plank2	Plank3	Bridge L1	Bridge L 2	Bridge L3	Bridge R1	Bridge R2
240.00	106.43	190.06	193.54	59.59	66.87	106.00	54.64	55.02
212.00	108.17	165.45	170.02	56.47	76.09	67.00	72.16	108.82
280.00	94.21	128.04	138.04	55.04	68.41	75.00	73.07	107.26
229.00	78.64	92.83	118.66	55.58	66.23	57.36	63.48	90.84
276.00	107.95	138.06	148.98	40.85	83.99	100.44	48.91	103.81
205.00	91.05	99.93	110.51	19.04	61.28	49.32	43.67	53.18
203.00	106.27	125.43	138.94	43.88	59.67	81.12	60.99	69.41
173.00	48.83	54.01	69.00	39.94	59.10	60.12	36.69	60.44
168.00	48.47	96.89	105.89	23.06	30.13	37.82	23.86	41.88
251.00	72.37	83.21	90.98	29.06	37.79	39.99	33.83	40.27
250.00	61.05	87.23	92.23	35.30	41.16	47.86	63.95	70.51
239.00	73.86	89.87	92.29	44.91	46.43	50.37	53.00	60.57
243.00	88.96	88.21	92.66	40.05	44.51	45.36	48.69	52.60
243.00	64.37	78.21	70.82	32.93	35.97	36.21	49.97	50.20
160.00	44.79	50.89	49.04	35.51	40.22	45.31	52.85	50.66
169.00	35.93	46.21	50.01	29.07	36.87	39.43	38.11	42.44
190.00	104.29	120.14	127.99	59.08	71.77	78.16	40.28	47.93
184.00	38.09	58.17	58.17	37.05	42.61	45.52	34.90	40.29

Bridge R3	sidestep1	sidestep2	sidestep3	30m1	30m2	30m3	400m1	400m2
70.00	32.00	30.00	34.00	5.16	5.00	4.61	72.74	70.94
110.00	31.00	30.00	32.00	5.45	5.38	4.85	73.95	75.87
100.00	34.00	34.00	38.00	4.88	4.93	4.42	66.71	61.59
87.39	33.00	34.00	37.00	5.04	4.73	4.62	73.16	71.73
110.16	32.00	34.00	34.00	5.01	4.28	4.30	66.86	59.76
77.64	24.00	26.00	33.00	4.63	4.68	5.37	82.09	81.73
80.98	30.00	31.00	33.00	6.16	5.56	5.44	93.02	90.87
59.00	26.00	26.00	28.00	6.95	6.25	6.16	113.57	100.78
47.03	26.00	28.00	30.00	7.36	6.17	6.02	116.57	98.92
43.70	32.00	31.00	31.00	4.84	4.83	4.81	68.73	69.79
72.25	32.00	30.00	32.00	5.27	5.08	5.09	68.26	78.71
63.92	32.00	30.00	34.00	5.45	5.04	5.06	85.72	87.03
51.39	34.00	34.00	33.00	4.67	4.79	4.78	87.14	85.60
53.98	30.00	29.00	28.00	5.86	5.59	5.70	98.61	96.20
53.85	26.00	25.00	24.00	7.51	7.23	6.98	106.81	107.72
45.98	30.00	30.00	29.00	6.94	6.87	6.83	113.84	114.63
46.47	26.00	26.00	26.00	6.35	6.13	6.12	104.33	98.02
45.14	27.00	27.00	28.00	6.60	6.17	6.12	110.48	97.81

400m3	YBTleft-A	YBT left-A	YBT left-A	YBT left-P	YBT left-P	YBT left-P	YBTleft-P	YBT left-P
71.31	54.00	68.00	72.00	70.00	84.00	95.00	88.00	90.00
76.03	52.00	64.00	66.00	102.00	98.00	110.00	100.00	100.00
61.23	68.00	76.00	78.00	108.00	103.00	114.00	96.00	97.00
71.05	60.00	78.00	77.00	104.00	106.00	109.00	90.00	93.00
58.29	88.00	100.00	103.00	88.00	106.00	119.00	96.00	101.00
82.00	44.00	60.00	59.00	90.00	81.00	96.00	78.00	87.00
91.48	66.00	70.00	72.00	88.00	94.00	104.00	92.00	93.00
101.00	66.00	78.00	80.00	76.00	86.00	98.00	90.00	91.00
99.62	54.00	64.00	66.00	84.00	86.00	94.00	87.00	86.00
69.89	78.00	90.00	85.00	82.00	80.00	89.00	82.00	85.00
78.02	86.00	88.00	90.00	114.00	110.00	103.00	114.00	119.00
87.41	56.00	62.00	62.00	92.00	88.00	96.00	78.00	79.00
86.01	56.00	60.00	59.00	80.00	75.00	83.00	88.00	83.00
96.03	48.00	50.00	52.00	84.00	88.00	90.00	58.00	59.00
106.98	52.00	51.00	52.00	74.00	75.00	80.00	82.00	83.00
114.23	62.00	63.00	61.00	94.00	96.00	87.00	90.00	88.00
96.30	64.00	54.00	59.00	78.00	84.00	82.00	82.00	82.00
96.30	68.00	70.00	72.00	78.00	84.00	80.00	80.00	81.00

YBT left-P	YBT right-A	YBT right-	YBT right-	YBT Right	YBT Right	YBT Right	YBT Right	YBT Right
104.00	54.00	62.00	63.00	100.00	109.00	110.00	86.00	98.00
112.00	54.00	65.00	65.00	101.00	108.00	108.00	94.00	105.00
105.00	68.00	82.00	82.00	102.00	110.00	113.00	94.00	100.00
103.00	60.00	85.00	84.00	104.00	106.00	108.00	95.00	100.00
109.00	74.00	75.00	76.00	100.00	107.00	110.00	80.00	91.00
90.00	40.00	66.00	67.00	90.00	97.00	97.00	72.00	79.00
94.00	64.00	66.00	70.00	82.00	91.00	98.00	66.00	97.00
91.00	58.00	68.00	66.00	80.00	95.00	100.00	76.00	94.00
98.00	52.00	68.00	67.00	82.00	94.00	96.00	78.00	89.00
92.00	76.00	70.00	71.00	98.00	97.00	93.00	80.00	79.00
120.00	90.00	90.00	90.00	118.00	120.00	124.00	106.00	105.00
87.00	60.00	61.00	59.00	98.00	96.00	94.00	90.00	87.00
84.00	52.00	51.00	53.00	98.00	87.00	89.00	88.00	79.00
62.00	48.00	49.00	47.00	74.00	75.00	70.00	62.00	63.00
84.00	50.00	53.00	52.00	84.00	88.00	85.00	82.00	79.00
89.00	56.00	55.00	52.00	90.00	87.00	87.00	84.00	77.00
80.00	72.00	73.00	72.00	98.00	93.00	98.00	82.00	80.00
85.00	62.00	60.00	58.00	92.00	92.00	90.00	80.00	83.00

YBT Right-PM3
99.00
107.00
103.00
105.00
108.00
88.00
97.00
96.00
90.00
76.00
103.00
87.00
78.00
61.00
80.00
80.00
80.00
82.00
